# Effect of Fructooligosaccharides Supplementation on the Gut Microbiota in Human: A Systematic Review and Meta-Analysis

**DOI:** 10.3390/nu14163298

**Published:** 2022-08-12

**Authors:** Yuqi Dou, Xue Yu, Yuanli Luo, Botian Chen, Defu Ma, Jing Zhu

**Affiliations:** 1 School of Public Health, Peking University Health Science Center, Beijing 100191, China; 2 West China School of Public Health, Sichuan University, Chengdu 610041, China; 3 Institute of Biotechnology and Health, Beijing Academy of Science and Technology, Beijing 100089, China

**Keywords:** Fructooligosaccharides, gut microbiota, *Bifidobacterium* spp., gastrointestinal symptoms

## Abstract

**Background:** Numerous studies have investigated the effects of the supplementation of fructooligosaccharides (FOS) on the number of bacteria in the gut that are good for health, but the results have been inconsistent. Additionally, due to its high fermentability, supplementation of FOS may be associated with adverse gastrointestinal symptoms such as bloating and flatulence. Therefore, we assessed the effects of FOS interventions on the composition of gut microbiota and gastrointestinal symptoms in a systematic review and meta-analysis. **Design:** All randomized controlled trials published before 10 July 2022 that investigated the effects of FOS supplementation on the human gut microbiota composition and gastrointestinal symptoms and met the selection criteria were included in this study. Using fixed or random-effects models, the means and standard deviations of the differences between the two groups before and after the intervention were combined into weighted mean differences using 95% confidence intervals (CIs). **Results:** Eight studies containing 213 FOS supplements and 175 controls remained in this meta-analysis. *Bifidobacterium* spp. counts significantly increased during FOS ingestion (0.579, 95% CI: 0.444–0.714) in comparison with that of the control group. Subgroup analysis showed greater variation in *Bifidobacterium* spp. in adults (0.861, 95% CI: 0.614–1.108) than in infants (0.458, 95% CI: 0.297–0.619). The increase in *Bifidobacterium* spp. counts were greater in the group with an intervention duration greater than 4 weeks (0.841, 95% CI: 0.436–1.247) than an intervention time less than or equal to four weeks (0.532, 95% CI: 0.370–0.694), and in the group with intervention doses > 5 g (1.116, 95% CI: 0.685–1.546) the counts were higher than those with doses ≤ 5 g (0.521, 95% CI: 0.379–0.663). No differences in effect were found between FOS intervention and comparators in regard to the abundance of other prespecified bacteria or adverse gastrointestinal symptoms. **Conclusions:** This is the first meta-analysis to explore the effect of FOS on gut microbiota and to evaluate the adverse effects of FOS intake on the gastrointestinal tract. FOS supplementation could increase the number of colonic *Bifidobacterium* spp. while higher dose (7.5–15 g/d) and longer duration (>4 weeks) showed more distinct effects and was well tolerated.

## 1. Introduction

The human large intestine contains a large number of diverse bacterial flora, which is important for human health [1]. This essentially anaerobic microbiota is involved in a variety of beneficial host functions, including fermentation of undigested nutrients [2,3], synthesis of vitamins [4], and interaction with the immune system [5,6]. It is capable of converting undigested carbohydrates and proteins into short-chain fatty acids (SCFAs) through bacterial fermentation, which are then absorbed and released as energy for the host [7]. Nevertheless, not all gut bacteria are advantageous to health. Beneficial bacterial genera include *Bifidobacterium* spp. and *Lactobacillus* spp. [8,9], both of which are glycolytic, while potentially infective bacterial species (opportunistic pathogens), for instance, *Bacteroides* [10] and *Enterobacteriaceae* [11] are sometimes considered to be harmful. Several types of dietary fiber have been found to alter bacterial populations and metabolism in the gut microbiota in response to diet [12].

A lower-molecular-weight version of inulin called fructooligosaccharides (FOS) is found in perennial plants such as artichokes, chicory, onions, leeks, garlic, and asparagus, along with small amounts in cereals [13], and the highest concentration of FOS has been found in yacon [14]. Inulin is mostly a linear polymer of fructose, with glucose being the terminal sugar. Commercially, FOS is produced in either of two ways: by partially hydrolyzing inulin using endoglycosidases Raftilose from chicory inulin, or by synthesizing sucrose using fungal fructosidases, as in Neosugar [15].

In 1995, Gibson and Roberfroid defined prebiotics as a class of compounds that activate beneficial bacteria in the colon (*Lactobacillus* spp. and/or *Bifidobacterium* spp.) to improve health. Currently, a wide range of carbohydrate- and non-carbohydrate-rich substances are included in this definition [16]. The small intestine does not digest FOS, which is an oligosaccharide fiber, but is primarily utilized as a fuel source by colonic bacteria such as *Bifidobacterium* spp. [17]. These properties make it a prebiotic [7,18,19]. For in vitro culture of human fecal bacteria, studies have shown that FOS selectively stimulates *Bifidobacterium* spp. growth while maintaining probable pathogens such as *clostridia* at a low level [20].

However, it remains a matter of interest whether the addition of FOS to a normal diet results in beneficial alterations in the gut microbiota. According to research by Euler et al. [21], after supplementation in healthy term infants, bifidobacterial counts were statistically higher (*p* < 0.045) in the 1.5 g/L FOS formula group compared to the human milk-fed or 3.0 g/L FOS-formula groups. However, seven days after the end of the FOS supplementation, there were no appreciable variations in the amounts of *Lactobacillus* spp. and/or *Bifidobacterium* spp. in the treatment groups. A study by Souza et al. [22] found higher numbers of *Bifidobacterium* spp. in the FOS group compared to controls after a 4-week intervention in infants (*p* = 0.006), but Xia et al.’s research [23] revealed no significant difference. In a study with adults, Bouhnik et al. [24] found that bifidobacterial concentrations ranged from 7.9 ± 0.5 to 9.1 ± 0.3 log cfu/g (*p* < 0.01) during FOS intake and went back to initial values by day 12 of the intervention (no significant difference); however, their other study showed an increased and statistically significant difference (*p* < 0.001) in the number of *bifidobacteria* during the FOS intervention [25]. The results for *Lactobacillus* spp. are also inconsistent, with the study by Tandon et al. [26] and Ten et al. [27] showing that FOS (especially at high doses) promoted the proliferation of *Lactobacillus* spp. compared to the control group; however, there are also research studies that show no significant differences [28,29].

Numerous studies have shown that FOS can increase the number of bacteria in the gut that are good for health, but not all of them have been consistent in their findings. Consequently, it is essential to further understand the impact of FOS intake at different doses on changes in human microbial populations. Furthermore, due to its high fermentability [30], FOS intake may be associated with negative gastrointestinal symptoms such as bloating and flatulence, so we also performed an analysis of adverse gastrointestinal symptoms.

## 2. Method

### 2.1. Eligibility and Search Strategy

We carried out this systematic review and meta-analysis under the guidelines of the Meta-Analysis of Randomized Controlled Trials in Epidemiology and Preferred Reporting Items for Systematic Reviews. This work has been registered in PROSPERO, the International Prospective Register of Systematic Reviews, under the registration number CRD42022312446.

Using the electronic databases of PubMed, Embase, and Cochrane Library, relevant peer review manuscripts (conference abstracts and unpublished studies not included) published before 10 July 2022 and written in English were included. The following MeSH terms, words, and phrases were used in the construction of the systematic search: Oligofructose or Oligofructan or Fructooligosaccharide* or Fructo-oligosaccharide*. Since gut microbiota can be divided by different levels from phylum to genus, there are too many free words associated with intestinal flora, therefore we did not perform a search for intestinal microorganisms and then combine intestinal flora with FOS. Two researchers searched and screened independently.

### 2.2. Selection Criteria and Quality Assessment

Randomized controlled trial studies including either randomized supplementation with FOS or controlled feeding trials with stool samples analyzed by culture, PCR, or other methods reporting the abundance of short-chain fatty acid-producing bacteria and gastrointestinal symptoms were included. There was no delimitation of doses and duration of FOS supplementation. Each aspect of the evaluation index, such as *Bifidobacterium* spp. in bacterial communities had to be explored in three or more studies, and each study provided mean and standard deviation values before and after the intervention before being included in this study. The units of colony counts are all log colony forming units per gram and gastrointestinal complaints were evaluated using a 0–10 scale or a 4-point Likert scale (none, mild, moderate, severe). Animal experiments or in vitro experiments, as well as intervention groups that were not or incompletely FOS (other prebiotics or inulin/oligofructose 50/50 mix) were excluded.

We evaluated the quality of the included studies using the Cochrane Collaboration Risk of Bias Tool [31], which includes the methods of random sequence generation, allocation concealment, blinding of participants and personnel, blinding of outcome assessment, incomplete outcome data, and selective reporting, where the symbols “−”, “?” and “+” stand for high, unclear, and low levels of bias, respectively.

### 2.3. Data Extraction

The data were extracted separately by two authors, who also independently evaluated the article’s quality. Any disagreements during this procedure were settled through conversation until agreement was obtained or by consulting a third author. Author, publication year, study nation, subject information, sample size, information about the intervention and control groups, RCT design, efficacy values, and 95% confidence intervals were among the information that was gathered.

### 2.4. Statistical Analysis

Cochran’s Q test was used to examine the statistical heterogeneity between the studies. The *I^2^* test was used to determine the degree of consistency (very low: <25%, low: 25–50%, moderate: 50–75%, large: >75%). DerSimonian–Laird (D-L) random effect modeling was used to estimate the weighted mean difference when the test for heterogeneity was statistically significant (*p* < 0.1 or *I*^2^ ≥ 50%). The supplementation and control groups’ pre- and post-intervention means and standard deviations were aggregated as weighted mean differences with a 95% confidence interval (CI).

Subgroup analysis was conducted based on the following variables: dose, duration, and intervention group. In addition, we performed a sensitivity analysis by removing one study from each round to calculate the impact of a single experiment on the overall outcome. To determine a linear dose/duration-response association between supplement dose, supplementation duration, and the intervention’s efficacy, a meta-regression analysis was utilized. The weighted mean differences of changes in the amount of increase in microbiota (*y*-axis) and supplement dose (*x*-axis) were shown.

The size of the circles indicates the weight of the included trials, which were calculated using the inverse of the total variance. The funnel Egger linear regression test and Begg’s rank correlation test were both used to assess the publication bias. We also investigated the publishing bias by visually examining funnel plots. If publication bias was identified, nonparametric analysis of publication bias was conducted using the trim and fill method.

All the analyses in this meta-analysis were performed using STATA (version 16.0; Stata Corp, College Station, TX, USA), and we considered a *p*-value of less than 0.05 (2-sided) to be statistically significant except a *p*-value of less than 0.10 for Cochran’s Q test.

## 3. Results

### 3.1. Literature Search and Study Characteristics

A preliminary electronic and manual literature search yielded a total of 2511 relevant abstracts of studies, of which 538 full texts were discarded due to repetition. After evaluating the titles, subjects, or abstracts of the remaining studies for eligibility and quality, 1943 papers were then excluded. The remaining 30 full-text studies were examined for a thorough assessment.

**Figure 1:**
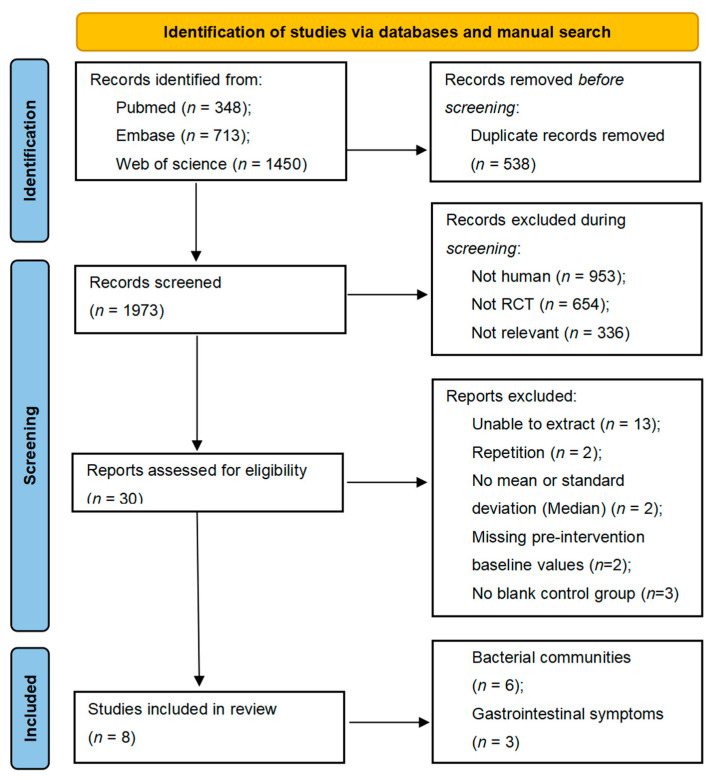
Flow chart of the included studies.

Of these, thirteen studies were excluded because relevant information could not be extracted (results were presented in graphs), two studies were excluded because they did not provide means and standard deviations of indicators but were presented as medians, and two studies were excluded because of duplication of the study populations. In addition, two studies without baseline information and three studies without blank controls were excluded. Considering the stringent inclusion and exclusion requirements, eight studies containing 213 FOS supplements and 175 controls remained in this meta-analysis. These included six studies of changes in flora counts [7,17,24,28,29,32,33] and three studies of gastrointestinal symptoms [29,34,35]. The details of the screened literature are displayed in Figure 1.

Table 1 displays the fundamental details of the included literature. The eight studies that passed the requirements for inclusion in this meta-analysis were published between 1995 and 2020. Of the eight studies, three of them were undertaken in France, two studies were implemented in the US, and two other studies were implemented in the UK. Two of the studies involved interventions with infants and the remaining six were adult studies. FOS supplementation doses ranged from 2.5 g/d to 15 g/d, and intervention duration was between 7 days and 56 days.

Sequence generation was at low risk in half of the studies. The danger of selection bias was unclear in 7 out of the 8 studies that omitted to disclose the allocation concealment strategy. Regarding performance bias, seven of the eight studies had a low risk of bias due to proper participant and staff blinding methods. Additionally, one study possessed a significant risk of attrition bias, whereas another contained an unclear risk of detection bias. There was little concern regarding reporting bias in any of the trials. The results are presented in the Supplementary Materials, Figure S1.

### 3.2. Changes in Gut Microbiota before and after Supplementation

**Figure 2:**
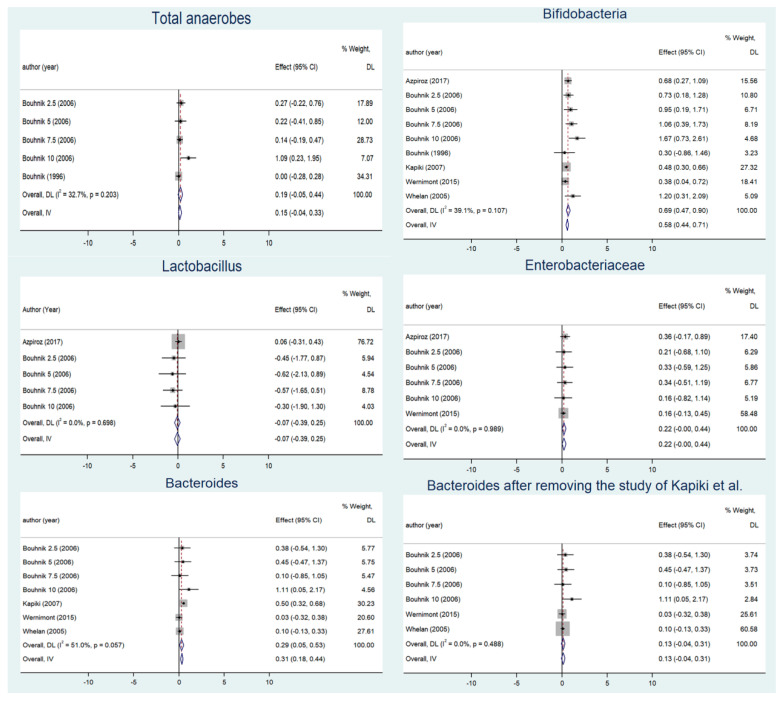
Summary analysis of changes in intestinal flora before and after supplementation [17,24,28,29,32,33].

The merge analysis is shown in Figure 2. Significant differences between the FOS groups and the placebo groups were not observed for counts of *Enterobacteriaceae* (0.220, 95% CI: −0.002–0.442, *P*_effect =_ 0.052; Q = 0.57, *I*^2^ = 0, *P*_heterogeneity_ = 0.989) or *Lactobacillus* spp. (−0.071, 95% CI: −0.392–0.250, *P*_effect_ = 0.665; Q = 2.21, *I*^2^ = 0, *P*_heterogeneity_ = 0.698). However, the counts of *Bifidobacterium* spp. dramatically increased as FOS was consumed (0.579, 95% CI: 0.444–0.714, *P*_effect_ < 0.001; Q = 13.14, *I*^2^ = 39.1%, *P*_heterogeneity_ = 0.107). As total anaerobes did not change over the intervention periods (0.148, 95% CI: −0.035–0.332, *P*_effect_ = 0.113; Q = 5.94, *I*^2^ = 32.7, *P*_heterogeneity_ = 0.203), *Bifidobacterium* spp. increased both in absolute numbers and as a proportion of total anaerobes. Subgroup analysis showed greater variation in *Bifidobacterium* spp. in adults (0.861, 95% CI: 0.614–1.108, *P*_effect_ < 0.001; Q = 5.68, *I*^2^ = 0, *P*_heterogeneity_ = 0.460) than in infants (0.458, 95% CI: 0.297–0.619, *P*_effect_ < 0.001; Q = 0.25, *I*^2^ = 0, *P*_heterogeneity_ = 0.615), and in those taking doses > 5 g (1.116, 95% CI: 0.685–1.546, *P*_effect_ < 0.001; Q = 3.31, *I*^2^ = 9.3, *P*_heterogeneity_ = 0.347) than those taking doses ≤ 5 g (0.521, 95% CI: 0.379–0.663, *P*_effect_ < 0.001; Q = 3.22, *I*^2^ = 0, *P*_heterogeneity_ = 0.522). Subgroup analysis of the intervention duration showed that the increase in *Bifidobacterium* spp. counts was higher in the group whose intervention lasted longer than four weeks (0.841, 95% CI: 0.436–1.247, *P*_effect_ < 0.001; Q = 8.97, *I*^2^ = 55.4%, *P*_heterogeneity_ = 0.062) than an intervention time less than or equal to four weeks (0.532, 95% CI: 0.370–0.694, *P*_effect_ < 0.001; Q = 3.12, *I*^2^ = 3.8, *P*_heterogeneity_ = 0.374) (Table 2). There was no change in the results of the sensitivity analysis (Supplementary Materials, Figure S2).

As for *Bacteroides*, the overall result showed that there is an increased trend after intervention (0.289, 95% CI: 0.048–0.530, *P*_effect =_ 0.019; Q = 12.25, *I*^2^ = 51%, *P*_heterogeneity_ = 0.057). Subgroup analysis revealed that the number of *Bacteroides* increased significantly when the doses were ≤5 g (0.330, 95% CI: 0.033–0.628, *P*_effect_ = 0.030; Q = 5.39, *I*^2^ = 44.4%, *P*_heterogeneity_ = 0.145); however, it was not significant when the dose was >5 g (0.277, 95% CI: −0.233–0.787, *P*_effect_ = 0.287; Q = 3.37, *I*^2^ = 40.6%, *P*_heterogeneity_ = 0.186). In addition to this, the change in the colony count of *Bacteroides* was also insignificant, both in adults and in infants, regardless of the duration of the intervention. A sensitivity analysis suggested that the Kapiki et al. study (a shorter intervention time) had a significant impact, and when this study was removed, neither the results of the pooled analysis (0.134, 95% CI: −0.044–0.312, *P*_effect_ = 0.139; Q = 4.44, *I*^2^ = 0%, *P*_heterogeneity_ = 0.488) nor the results of the subgroup analysis were significant.

### 3.3. Adverse Gastrointestinal Reactions before and after Supplementation

**Figure 3:**
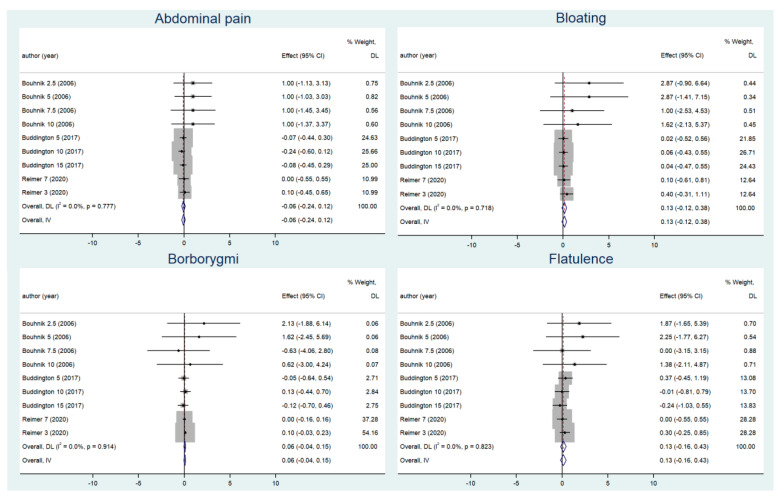
Changes in adverse gastrointestinal reactions before and after supplementation [29,34,35].

As for gastrointestinal symptoms (Figure 3), the pooled results for borborygmi (0.055, 95% CI: −0.041–0.152, *P*_effect =_ 0.261; Q = 3.30, *I*^2^ = 0, *P*_heterogeneity_ = 0.914), bloating (0.128, 95% CI: −0.123–0.379, *P*_effect =_ 0.317; Q = 5.37, *I*^2^ = 0, *P*_heterogeneity_ = 0.718), abdominal pain (−0.061, 95% CI: −0.244–0.123, *P*_effect =_ 0.519; Q = 4.82, *I*^2^ = 0, *P*_heterogeneity_ = 0.777) and flatulence (0.134, 95% CI: −0.161–0.429, *P*_effect =_ 0.374; Q = 4.36, *I*^2^ = 0, *P*_heterogeneity_ = 0.823) were not significant and indicated that FOS intake did not increase these symptoms. Subgroup analyses on intervention dose had similar results to the pooled analyses, none of which were statistically significant (Supplementary Materials, Figure S3).

### 3.4. Meta-Regression

A meta-regression analysis was carried out to determine whether there was a linear dose–response or linear duration–response relationship between the supplementation dose, the duration of the intervention, and the effects on the changes in gut microbiota before and after supplementation. However, our research did not reveal any statistical significance (*P*_dose_ = 0.244) (*P*_duration_ = 0.760) (Supplementary Materials, Figures S4 and S5).

### 3.5. Publication Bias

To assess any potential publication bias, we used the Egger’s and Begg’s tests. The results were as followed. Total anaerobes according to the Egger’s and Begg’s tests were *p* = 0.043 and *p* = 0.086, respectively; *Bifidobacterium* spp. were *p* = 0.026 and *p* = 0.048, respectively; *Lactobacillus* spp. were *p* = 0.026 and *p* = 1, respectively; *Enterobacteriaceae* were *p* = 0.262 and *p* = 0.707, respectively; and *Bacteroides* were *p* = 0.113 and *p* = 0.133, respectively. The funnel plots are shown in the Supplementary Materials, Figure S6. The results after filling using the trim and filling method showed that the publication offset did not affect the summary results. It was still concluded that FOS supplementation significantly increased the number of *Bifidobacterium* spp., but did not affect other species.

## 4. Discussion

The evidence regarding the effects of FOS supplementation on the human gut flora was evaluated in our systematic review. Short-term FOS supplementation interventions were unlikely to cause changes in total anaerobic bacteria, but internal flora composition ratios were altered. At the genus level, the increase in fecal *Bifidobacterium* spp. concentration was greater in individuals supplemented with FOS than in individuals without FOS supplementation, whereas we did not observe changes in *Lactobacillus* spp. In the meanwhile, the number of potential pathogens, *Bacteroides* did not decrease with FOS supplementation. At the family level, the number of opportunistic pathogens, *Enterobacteriaceae* did not change significantly when consuming FOS. Our findings supported the selectivity criterion in the prebiotic concept that FOS was used as a selective substrate by host microbes, which may contribute to the host’s health [36].

As typical prebiotics, FOS has been used extensively to promote the growth of *Bifidobacterium* spp. [37], and in certain situations, *Lactobacillus* spp. [38]. With a significant impact on *Bifidobacterium* spp. but no effect on *Lactobacillus* spp., the FOS intervention in our investigation had a varied impact on the abundance of the two genera. This may indicate that the two genera have different preferences for substrates, with *Bifidobacterium* spp. being less able to distinguish between different fermentation substrates than *Lactobacillus* spp. [39,40]. The β-fructosidase expressed in *Bifidobacterium* spp. is rather abundant and selective for the β1–2 glycosidic linkages present in FOS [41,42]. Subsequent transport mechanisms and hydrolysis rates may also be faster. The monomer then functions as a productive growth substrate for the Bifidus pathway following oligosaccharide hydrolysis [7].

Infants that are breastfed typically have a gut microbiota dominated by *Bifidobacterium* spp., which has advantageous properties [43]. They exist as a result of the diverse constituents of breast milk, which include prebiotic substances [32]. The addition of small amounts of FOS to bottle-fed preterm or term infants was well tolerated and resulted in similar number of Bifidobacterium spp. as breastfed infants and an increase in the number of Bifidobacterium spp. compared to normal formula [17]. In addition to this, the results of the subgroup analysis showed that the number of *Bifidobacterium* spp. increased more in the group with a long intervention time (>4 weeks) and more in the high intervention dose group (>5 g) than in the group with a short intervention time (≤4 weeks) and a low intervention dose (≤5 g), but the trend was not significant by meta-regression due to the limited number of studies in the of literature.

Bacteroidetes appear to make up around a quarter of the entire bacterial population, according to molecular analysis techniques based on PCR and fluorescence in situ hybridization (FISH) [44]. Gram-negative, obligate anaerobic bacteria belonging to the genus *Bacteroides* use the fermentation of various plant-derived sugar compounds as their primary source of energy. These potentially hazardous substances are prevalent in the human colon. These sugars are transformed into advantageous fermentation products by *Bacteroides* such as *B. thetaiotaomicron* [45]. *Bacteroides* can also take the side chains from bile acids, and reintroduce them into the hepatic circulation [46]. Some species, such as *B. fragilis*, are opportunistic human pathogens that can cause appendicitis, gastrointestinal surgery, and abdominal infections by forming abscesses, blocking phagocytosis, and inactivating β-lactam antibiotics [47,48]. Unlike the pooled results of the inulin intervention [49], our study showed that the FOS intervention did not result in a reduction in the number of *Bacteroides*.

Many of the more well-known pathogens, such as *Salmonella*, *Escherichia coli*, *Klebsiella*, and *Shigella*, are members of the broad family of Gram-negative bacteria known as *Enterobacteriaceae*. This family also contains a huge number of unharmed symbionts and the pathogens, *Enterobacter* and *Citrobacter*. These pathogens have the potential to cause or contribute to human diseases such as intra-abdominal infections and bacteremia [50]. Prebiotic therapies have been demonstrated to be effective in lowering the prevalence of some opportunistic infections, including *Enterobacter* [38]. Since the three included studies were explored at the family level without subdividing the individual genera, we cannot know the situation at the genus level; however, at the family level, there was no significant change before and after the intervention.

This study verified that FOS is well tolerated in a range of doses. Human prebiotic feeding studies have found many reports of symptoms related to gas production in the gut, but they are still only extremely moderate at the suggested intake levels [7,51]. We found no discernible symptoms of gastric discomfort when compared to placebo. Similarly, GI symptoms showed no dose–response relationship.

The strengths of the current study are that it included all randomized controlled trial designs, and by evaluating a supplement containing a single prebiotic (FOS) that was not used in combination with other prebiotics like galacto-oligosaccharides or inulin, the study was able to specifically assess the impact of FOS on fecal microbiota as well as on symptoms of gastrointestinal distress.

There are several limitations that should be mentioned. First, four of the eight studies included did not describe in detail how the random sequences were generated, and six did not describe in detail the implementation of allocation concealment, so it was not possible to determine the selection bias; second, the methods of colony detection were not the same, and differences in detection methods can lead to differences in results; third, due to limitations in the number of studies, we could not explore the dose and duration thresholds of FOS supplementation on *Bifidobacterium* spp., and detailed analysis of the potentially pathogenic species (*Enterobacteriaceae*) was not possible; and finally, intestinal bacteria offer FOS as an easily fermentable fiber, producing significant amounts of SCFAs [52]. To understand the role of FOS, besides focusing on the changes in intestinal flora, its metabolites, SCFAs, are also the elements to focus on. The limited number of taxa analyzed in the review may not accurately reflect the total impact of FOS interventions on gut microbiota composition and metabolic outputs. In light of this, follow-up studies need to be further explored.

Future randomized controlled trials should examine how FOS supplementation affects the human gut microbiome and its health implications, while taking into account the baseline gut microbiota composition (for example, enterotype) and the dietary characteristics of participants to pinpoint the exact effects of FOS. Besides, metatranscriptomics, metaproteomics, and metabolomics could be included in future studies to better understand the effects of FOS supplementation. In addition, longer-term studies are also required to more accurately evaluate the long-term impact on microbial diversity.

## 5. Conclusions

In conclusion, FOS supplementation could increase the number of colonic Bifidobacterium spp. while higher dose (7.5–15 g/d) and longer duration (>4 weeks) showed more distinct effects and was well tolerated.

## Figures and Tables

**Table 1:** Description of included studies.

Study	Participants (N)	Interventions		RCT Design					Index of Analysis
		Compound Studied and Dose	Comparator	Design	Duration (d)	Run-in	Washout	Analysis (Microbiota)	
Azpiroz, 2017 [28]	France; IBS patients (34 scFOS group and 34 Placebo group); 18–60 years age	scFOS (Actilight 950P; Beghin_x0002_Meiji, Marckolsheim, France), 5 g per day	Maltodextrins (Maldex 120; Tereos Syral, Marckolsheim, France)	Parallel	28	×	×	real-time PCR; 16S rDNA	Intestinal flora
Bouhnik, 1996 [24]	France; Healthy volunteers (10 Fructo-Oligosaccharides and 10 Placebo); 22–39 years age	FOS (Actilight™, Eridania-Beghin Say, Paris, France), 12.5 g/day	Saccharose	Parallel	36	×	×	Culture	Intestinal flora
Bouhnik, 2006 [29]	France; Healthy volunteers (32 FOS and 8 placebo); 29 ± 1.3 years age	scFOS (Actilight™, Beghin Meiji, Paris, France); 2.5, 5.0, 7.5 or 10 g/d.	50% sucrose—50% fully digestible waxy maize-derived maltodextrins (DE6.5) (Cerestar, Vil_x0002_voorde, Belgium)	Parallel	7	√	×	Culture	Intestinal flora, gastrointestinal symptoms
Buddington, 2017 [34]	USA; Adults (49 OF and 48 placebo) having a body mass index ≤ 35; age: from 18 to 65 years	OF (Orafti^®^ P95 Oligofructose), 5 g/day, 10 g/day and 15 g/day	Maltodextrin	Parallel	28	√	×	/	Gastrointestinal symptoms
Gibson, 1995 [7]	UK; Eight healthy volunteers with a mean body mass index of 22.4; age: 21–48 years	Oligofructose (Orafti, Tienen, Belgium), 15 g/day	Sucrose	Crossover	15	×	√	Culture	Intestinal flora
Kapiki, 2007 [32]	Greece; Preterm infants (36 FOS and 20 placebo) with a maximum gestational age of 36 weeks	FOS, 0.4 g/100 mL	Maltodextrins	Parallel	7	×	×	Culture	Intestinal flora
Reimer, 2020 [35]	Canada; Healthy adults without obesity (BMI: 18.5–29.9) (11 Moderate dose ITF and 14 placebo or 11 Low dose ITF and 12 placebo); age: 18–65 years	Trial 1—Moderate Dose ITF snack bar, 7 g/d Trial 2—Low Dose ITF snack bar, 3 g/d	Control 1 snack bar Control 2 snack bar	Crossover	28	√	×	q-PCR; 16S rRNA	Gastrointestinal symptoms
Wernimont, 2015 [17]	USA; Infants (20 CF and 19 EF); 11.2 ± 2.3	OF (Orafti^®^ P95, Tienen, Belgium), 3.0 g/L	α-lactalbumin-enriched control formula (CF)	Parallel	56	×	×	FISH	Intestinal flora
Whelan, 2005 [33]	UK; Healthy men and women (*n* = 10) between 21 and 34 y old	FOS (Nutren fiber, Nestle’ Switzerland), 9.5 ± 1.5 g/d	Standard (FOS and fiber-free) enteral formula (Nutren 1.0, Nestle´ Switzerland)	Crossover	28	×	√	FISH	Intestinal flora

**Table 2:** Subgroup analysis of changes in intestinal flora before and after supplementation.

Gut Microbiota	Subgroup		WMD (95% CI)	Heterogeneity	
				*I* ^2^	*p* Value
*Bifidobacterium* spp.	dose	≤5 g	0.52 (0.38, 0.66 )	0	0.522
		>5 g	1.12 (0.69, 1.55)	9.3%	0.347
	duration	≤4 weeks	0.53 (0.37, 0.69)	3.8%	0.374
		>4 weeks	0.84 (0.44, 1.25)	55.%	0.062
	intervention group	adult	0.86 (0.61, 1.11)	0	0.460
		infant	0.46 (0.30, 0.62)	0	0.615
*Lactobacillus* spp.	dose	≤5 g	−0.01 (−0.35, 0.33)	0	0.550
		>5 g	−0.49 (−1.38, 0.41)	0	0.784
*Enterobacteriaceae*	dose	≤5 g	0.21 (−0.02, 0.45)	0	0.923
		>5 g	0.26 (−0.38, 0.90)	0	0.786
*Bacteroides*	dose	≤5 g	0.40 (0.24, 0.56)	44.4%	0.145
		>5 g	0.14 (−0.07, 0.36)	40.6%	0.186
	duration	≤4 weeks	0.31 (−0.09, 0.70)	86%	0.008
		>4 weeks	0.19 (−0.10, 0.47)	5.1%	0.377
	intervention group	adult	0.17 (−0.04, 0.38)	0	0.408
		infant	0.29 (−0.17, 0.75)	81.4	0.020
*Bacteroides* after removing the study of Kapiki et al.	dose	≤5 g	0.12 (−0.19, 0.43)	0	0.592
		>5 g	0.14 (−0.07, 0.36)	40.6%	0.186
Total anaerobes	dose	≤5 g	0.25 (−0.13, 0.64)	0	0.902
		>5 g	0.23 (−0.17, 0.63)	64.1%	0.062

## Supplementary Materials

The following supporting information can be downloaded at: https://www.mdpi.com/article/10.3390/nu14163298/s1, Figure S1: Risk of bias assessment for literatures included in this meta-analysis; Figure S2: Sensitivity analysis results for the changes in intestinal flora; Figure S3: Subgroup analysis of adverse gastrointestinal reactions; Figure S4: Meta-regression analysis of the relationship between the supplement dose and the changes in intestinal flora; Figure S5: Meta-regression analysis of the relationship between the supplement duration and the changes in intestinal flora; Figure S6: Funnel plots for the changes in intestinal flora.
