# Supplementary material for: Effect of Fructooligosaccharides Supplementation on the Gut Microbiota in Human: A Systematic Review and Meta-Analysis
Source: Nutrients. 2022 Aug 12;14(16):3298. doi: 10.3390/nu14163298 (PMC9413759; doi:10.3390/nu14163298)
Supplement: Supplementary file 1 [file nutrients-14-03298-s001.zip › nutrients-1810319-supplementary.pdf]

Supplementary Material

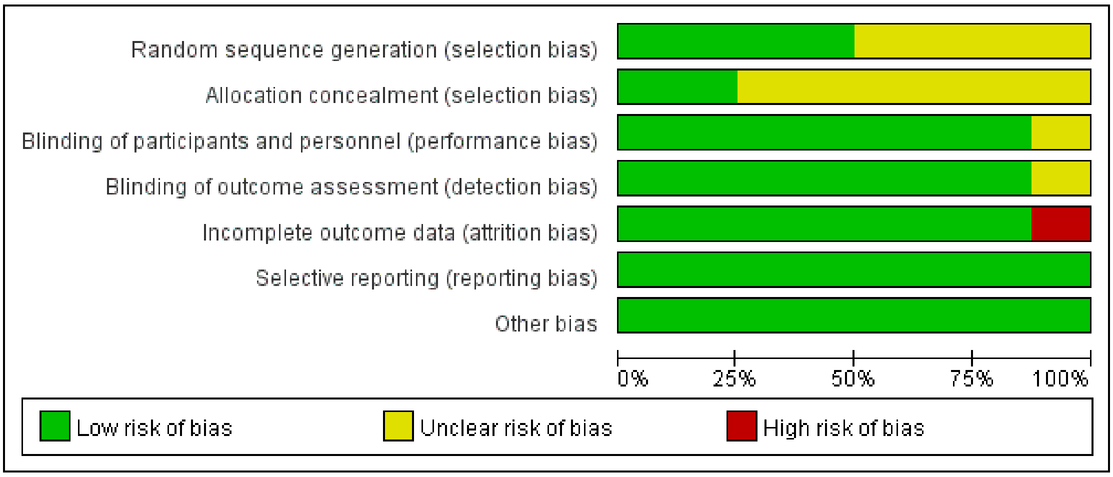

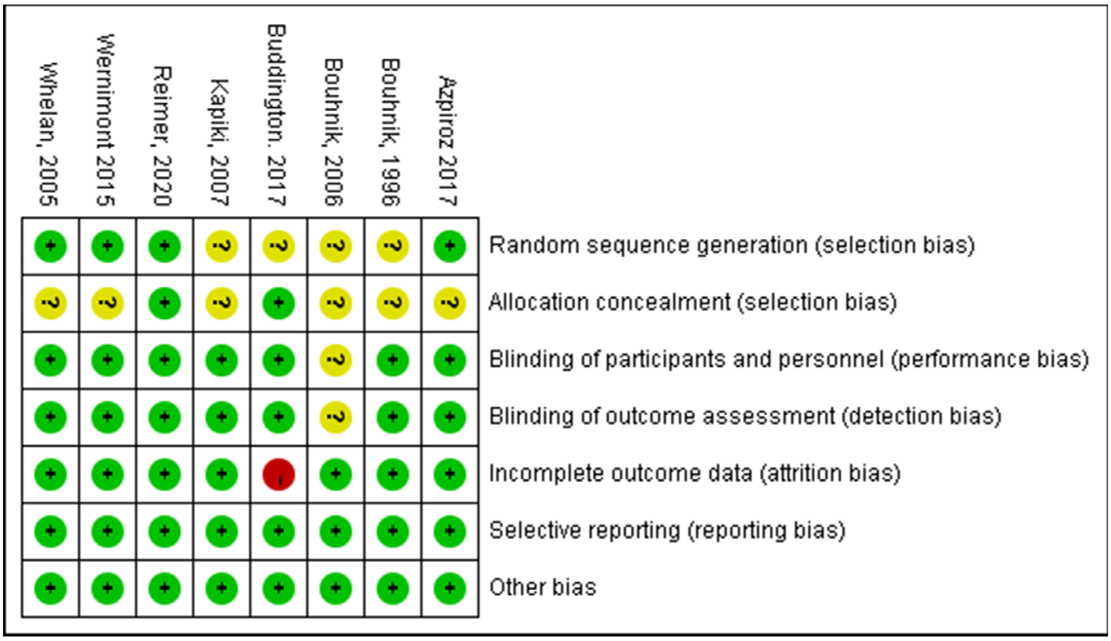

Figure S1. Risk of bias assessment for literatures included in this meta-analysis

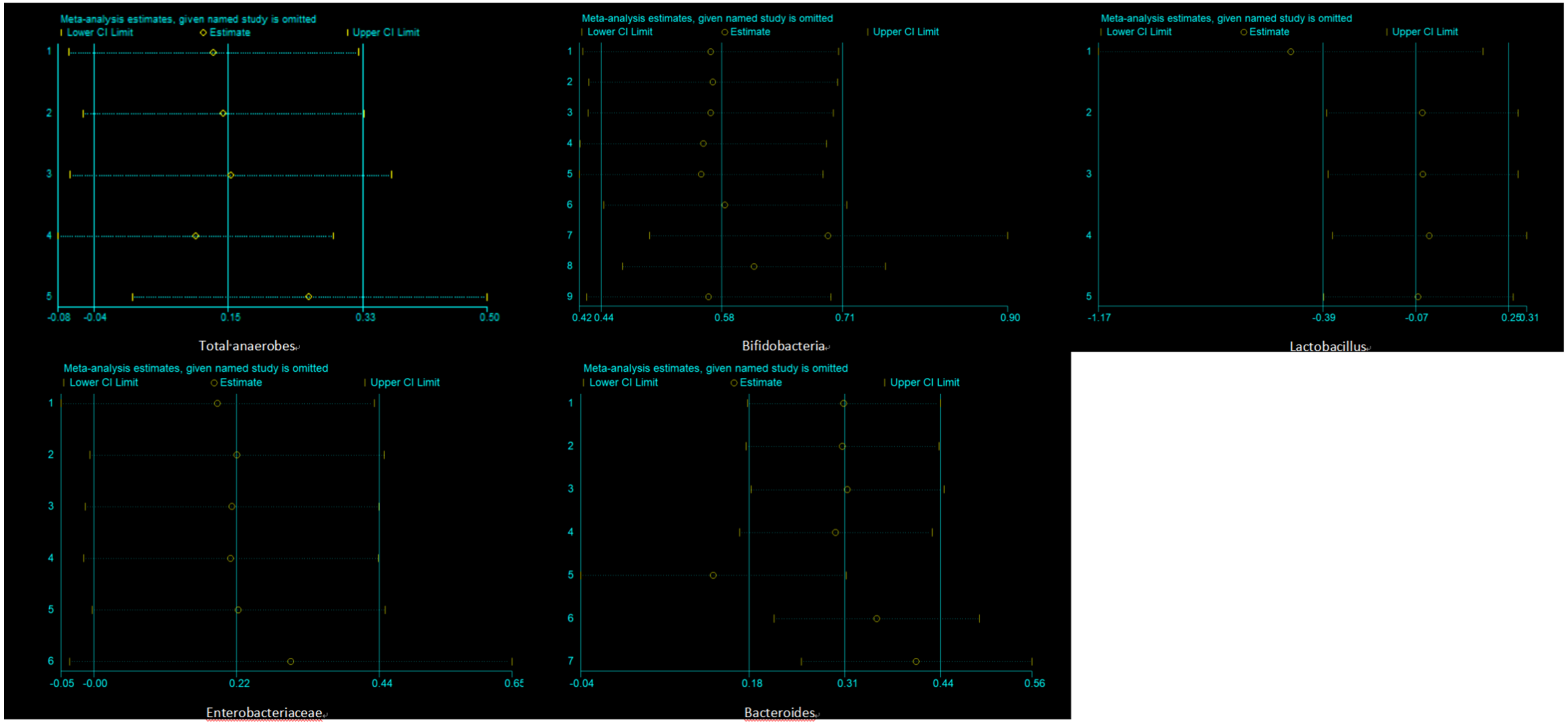

Figure S2. Sensitivity analysis results for the changes in intestinal flora.

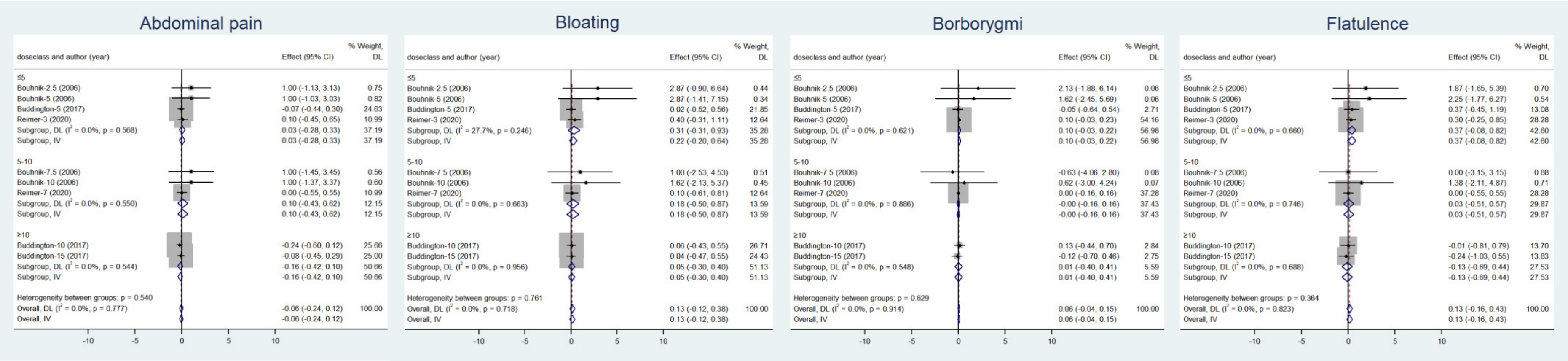

Figure S3. Subgroup analysis of adverse gastrointestinal reactions

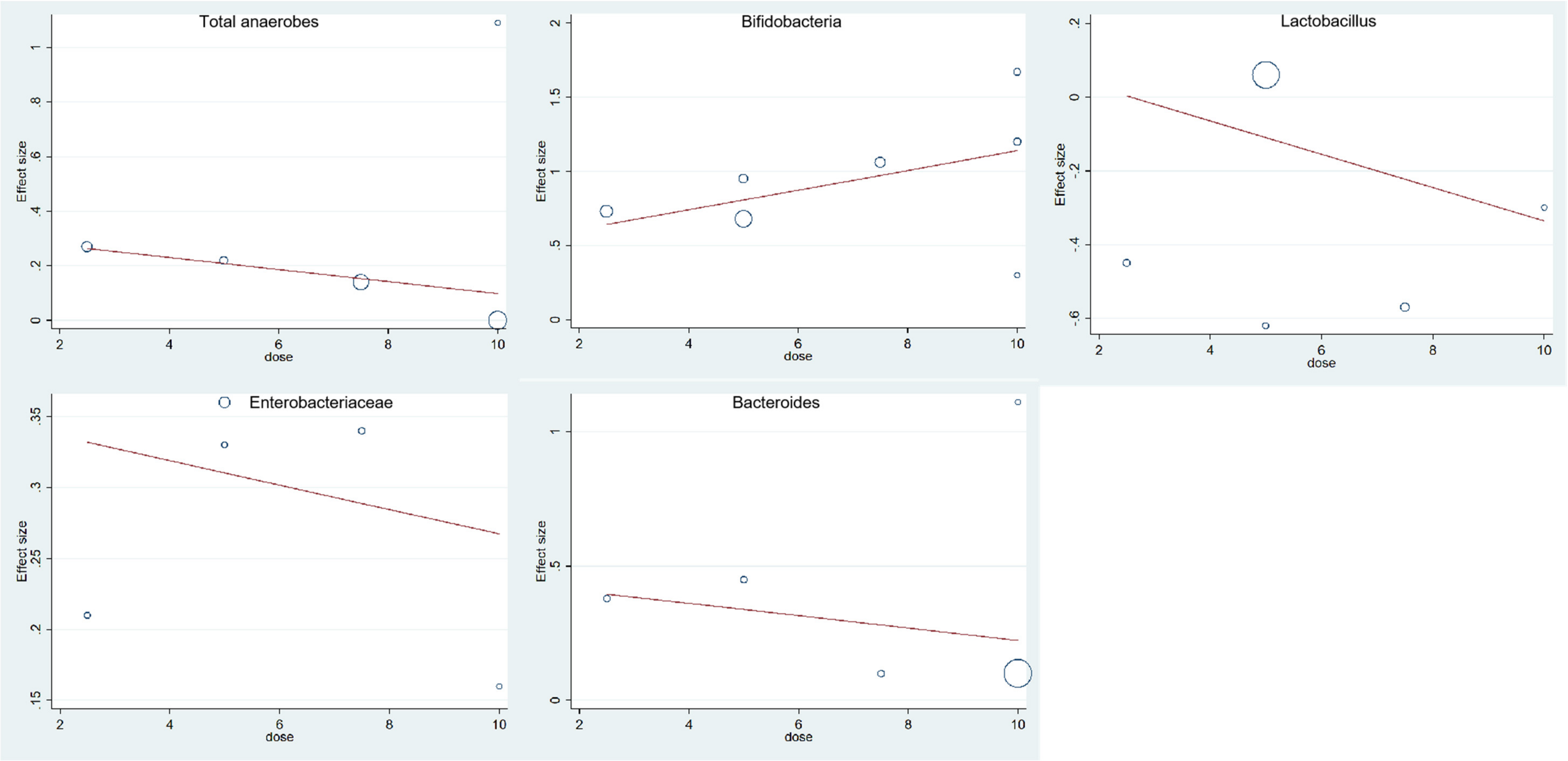

Figure S4. Meta-regression analysis of the relationship between the supplement dose and the changes in intestinal flora.

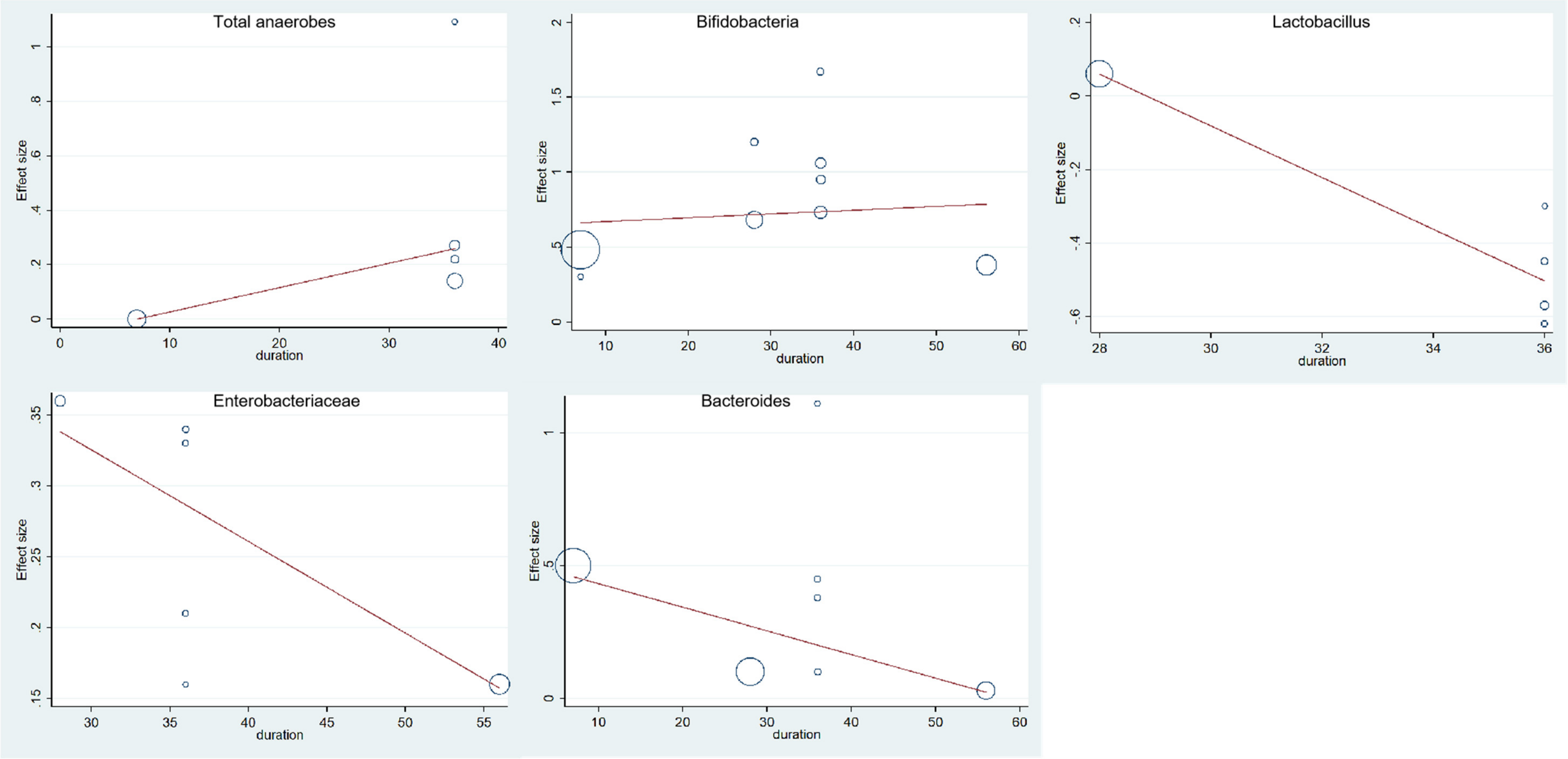

Figure S5. Meta-regression analysis of the relationship between the supplement duration and the changes in intestinal flora.

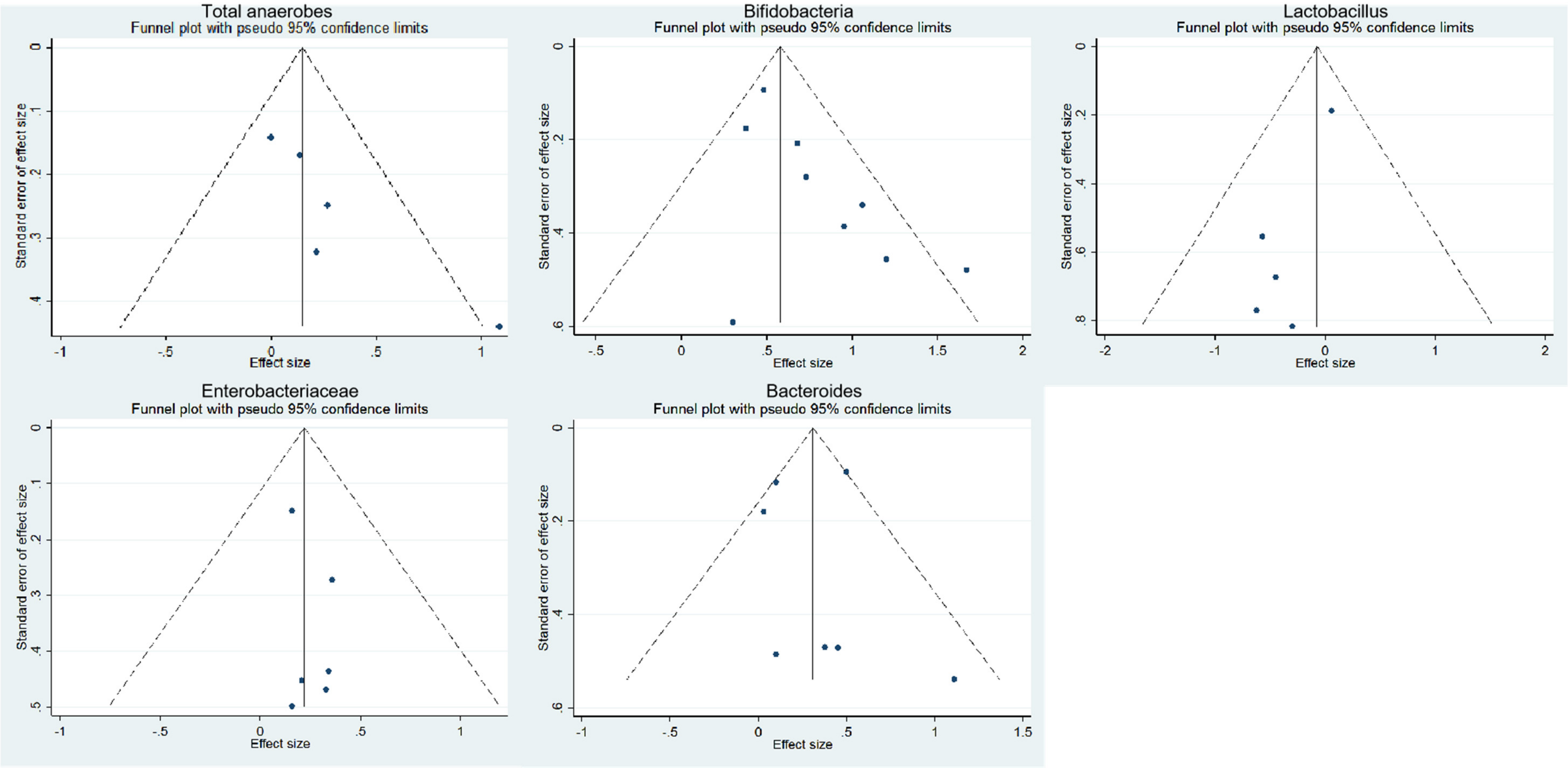

Figure S6. Funnel plots for the changes in intestinal flora.
